# The Influence of the Nervous System on the Health of the Mouth

**Published:** 1879-01

**Authors:** Worthington Myers

**Affiliations:** New York


### Selections.

### THE INFLUENCE OF THE NERVOUS SYSTEM ON THE HEALTH OF THE MOUTH.

BY WORTHINGTON MYERS, A. M., M. D., NEW YORK.

For some years I have been much interested in noticing the conditions of the nervous system in connection with the health or disease in the mouth of persons coming under my personal observation, and I will as briefly as possible, state some conclusions arrived at from such observations.

We are well aware that there are various degrees of nervous activity or restlessness, as well as weakness, also strength or tranquility, not only in different individuals, but frequently in the same individual at different times.

We also know that the health of the mouth varies in different persons, as well as in the same person at different times. Let us look at the extremes of each.

In regard to various mouths, they may be considered chemically, as in two classes, the alkaline and the acid. I allude to the general character of the fluids of the mouth, without reference to any diversity that may sometimes be found in its various secretions.

In the alkaline mouth we usually find it of more dense structure, and not easily affected by what are considered injurious agents, and only so affected when such agents find very obscure lurking places for some length of time. In this mouth the general character of its secretions is so decidedly alkaline that they act as a constant and effective neutralizing agent to any acid influence within their reach.

On the other hand, we find in the acid mouth, though the structures are less dense, that it is more easily affected by acid elements taken into, as well as readily developed in the month.

Now, if we observe, we shall find existing with these two extremes of condition corresponding extremes in the condition of the nervous system, which may be classed as tranquil or strong, and restless or active (or weak).

In the tranquil we find sufficient strength of nerve power to take the individual along in life with a comfortable feeling of reserved strength, usually attended by good digestion.

The nervous system may be constitutionally quiet or strong, or even stolid, or there may be such freedom, from continued care or close application that the nervous power is not actually tested or overdrawn.

But in the restless or active condition, we see perhaps, a constitutional disposition to activity of mind or body, or both; or else a state of health, or a continued pressure of circumstances that draws so unremittingly on the stock of nerve power that there is not sufficient opportunity to acquire a proper reserve of that power, often resulting in various uncomfortable sensations and not unfrequently attended by dyspepsia.

Besides some states of health and local conditions in the human organization that are known to produce this unsettled state of the nervous system, there are various other causes, some of whch perhaps, are necessary attendants on our civilization, that no doubt contribute most largely to its very general existence. Such are the constant cares and anxieties of business, social and domestic life, and the strong and continued pressure, through ambition or otherwise, imposed on the energies of growing youth during a large part of the year; and we daily see the effect of the various causes of over-work, as shown by their results in the mouths of patients.

My conclusions are, that where the nervous power is not unduly active or overdrawn, as a rule, the digestive system will be in such condition that the general character of the secretion of the mouth will be alkaline and corrective of acidity, which will likewise prove a source of destruction to the teeth. And, on the other hand, that where the nervous system *is* unduly active, or its powers overtasked for a length of time, we may expect to find the fluids of the mouth tending to an acid character in proportion to such overdraft.

Also, to reverse the rule, whenever we find its membranes strong and healthy—we may diagnose a tranquility of nerve; and whenever we find them in a flabby condition of a white and acid character, we may be equally sure that the nervous system is or has been for some time overtasked.

Of course, in practice we see all degrees between the extremes I have mentioned, but I am satisfied their proportions will be found to correspond, if close observation be given.

These conditions may exist under favoring circumstances, for a large part of one's lifetime, or they may vary at different periods of life and under different circumstances, or that we sometimes see a mouth which has always been healthy; but how much oftener do we see the contrary.

In general terms, then, the result of these observations has been to prove (other things being equal) that so far as tranquility of nerve prevails, so far we may expect soundness of the membranes of the mouth, and in proportion as the restless condition exists in continuance, in that degree will there be disease in the mouth.

Much might be said of the various evidences of this rule; and a correct appreciation of it will be found of great utility in enabling us to decide when and how to attempt the treatment of an organ which plays so important a part in the animal economy, and, which in a sound condition, contributes so much to the health and comfort, the happiness and comeliness of the human race.—*Lancet and Clinic.*

